# Effects of Infliximab in a Propionic Acid-Induced Experimental Autism Rat Model

**DOI:** 10.3390/biomedicines14040940

**Published:** 2026-04-20

**Authors:** Nur Akman, Ahmet Ufuk Kömüroğlu, Salih Çibuk, Fikret Altındağ, Osman Yılmaz, Ahmet Ateşşahin

**Affiliations:** 1 Department of Midwifery, Faculty of Health Sciences, Van Yuzuncu Yil University, 65080 Van, Türkiye; 2 Vocational School of Health Services, Van Yuzuncu Yil University, 65080 Van, Türkiye; aukomuroglu@yyu.edu.tr (A.U.K.);; 3 Department of Histology and Embryology, Division of Basic Medical Sciences, Faculty of Medicine, Van Yuzuncu Yil University, 65080 Van, Türkiye; 4 Department of Anatomy, Division of Basic Veterinary Sciences, Faculty of Veterinary Medicine, Van Yuzuncu Yil University, 65080 Van, Türkiye; 5 Department of Veterinary Pharmacology and Toxicology, Faculty of Veterinary Medicine, Fırat University, 23200 Elazig, Türkiye

**Keywords:** autism spectrum disorder, propionic acid, infliximab, neuroinflammation, glial activation, BDNF

## Abstract

**Background/Objectives**: Autism spectrum disorder (ASD) is a neurodevelopmental condition increasingly associated with dysregulated neuroimmune signaling and altered neurotrophic homeostasis. Tumor necrosis factor-alpha (TNF-α) has been implicated in ASD pathophysiology; however, the downstream effects of TNF-α blockade on cytokine–neurotrophin interactions during neurodevelopment remain insufficiently characterized. In this study, we evaluated the effects of infliximab (IFX), a monoclonal anti-TNF-α antibody, on behavioral performance, neuroinflammatory cytokine profiles, glial activation, and brain-derived neurotrophic factor (BDNF) signaling in a propionic acid (PPA)-induced experimental ASD rat model. **Methods**: Experimental ASD was induced by propionic acid administration in rats. Animals were divided into control and treatment groups. Behavioral performance was assessed using the Morris Water Maze, direct social interaction, and three-chamber sociability tests. Levels of TNF-α, interleukin-1 beta (IL-1β), interleukin-6 (IL-6), and BDNF were measured in serum, hippocampal, and cerebellar tissues. Microglial and astrocytic activation were evaluated using CD11 and GFAP immunohistochemistry. **Results**: PPA administration resulted in pronounced impairments in learning, memory, and social behaviors, accompanied by elevated proinflammatory cytokine levels, increased BDNF expression, and marked glial activation in the hippocampus and cerebellum. Although IFX treatment significantly reduced TNF-α levels in central tissues, it did not improve behavioral deficits and was associated with persistently elevated IL-1β and IL-6 levels, sustained glial reactivity, and further alterations in BDNF levels. **Conclusions**: These findings suggest that TNF-α suppression alone does not normalize the disrupted cytokine–neurotrophin axis and may differentially modulate BDNF-related neuroplastic signaling during development. In conclusion, this study indicates that non-selective TNF-α blockade during neurodevelopment fails to confer behavioral benefit in experimental ASD and highlights the importance of considering cytokine–BDNF pathway interactions when designing immunomodulatory strategies for neurodevelopmental disorders.

## 1. Introduction

Autism spectrum disorder (ASD) is a heterogeneous neurodevelopmental condition characterized by deficits in social interaction and communication, as well as repetitive and stereotyped behaviors [1]. Recent epidemiological data indicate that ASD affects approximately one in every 31 children worldwide, highlighting its growing impact as a major public health concern [2]. Although the etiology of ASD has not yet been fully elucidated, it is widely accepted that interactions between genetic susceptibility and environmental factors play a fundamental role in disease development [3]. Accumulating evidence over the past decade increasingly suggests that neuroinflammatory processes may represent a central component of ASD pathophysiology [4,5,6].

Propionic acid (PPA)-based animal models are widely used in experimental studies to investigate ASD-related neurobehavioral and neurobiological alterations [7]. PPA is a short-chain fatty acid produced in the mammalian gut as a metabolic byproduct of enteric bacteria, particularly species belonging to the *Clostridia* and *Desulfovibrio* genera, or through the intestinal fermentation of dietary components. As a weak organic acid, PPA readily crosses the gut–blood–brain barrier and has been reported to induce intracellular acidification, oxidative stress, and neuroinflammatory responses within the central nervous system [4,8]. Previous studies have shown that PPA administration is associated with reduced social interaction, increased repetitive behaviors, and impairments in learning and memory, accompanied by elevated proinflammatory cytokine levels and structural alterations in brain tissue [4,5,6]. Collectively, these characteristics indicate that the PPA model provides a valid and reliable experimental approach for examining the neuroinflammatory and neuroplastic components of ASD.

Neuroinflammatory alterations in ASD have been reported to be heterogeneously distributed across the brain, with certain regions exhibiting more pronounced changes than others. In this context, the hippocampus and cerebellum are among the brain regions in which structural, functional, and molecular abnormalities associated with ASD have been most consistently reported [9,10]. The hippocampus plays a central role in fundamental cognitive processes such as learning, memory, and cognitive flexibility, whereas the cerebellum is increasingly recognized as a critical component of cortico–subcortical networks involved not only in motor coordination but also in higher-order functions, including attention, executive function, social behavior, language, and emotional regulation [11].

Experimental and clinical studies have reported increased levels of proinflammatory cytokines, including tumor necrosis factor-alpha (TNF-α), interleukin-1 β (IL-1β), and interleukin-6 (IL-6), in various brain regions of individuals with ASD, particularly in the hippocampus and cerebellum [12,13]. Elevated levels of these cytokines have been closely associated with microglial activation, disruption of synaptic integrity, and neuronal functional alterations. While IL-1β and IL-6 contribute to synaptic plasticity and neuronal adaptation under physiological conditions, chronic and dysregulated increases in these cytokines have been reported to suppress long-term potentiation, reduce dendritic spine density, and adversely affect learning and memory processes [14,15].

Considering the role of neuroinflammation in the pathophysiology of ASD, TNF-α has been regarded as a potential therapeutic target. Infliximab (IFX), a TNF-α inhibitor, has been shown to exert anti-inflammatory and neuroprotective effects in various experimental models by suppressing inflammatory responses [16,17,18]. However, TNF-α is known to serve not only as a proinflammatory cytokine within the central nervous system but also to play essential physiological roles in synaptic plasticity, glial homeostasis, and neurodevelopmental balance. Accordingly, non-selective inhibition of TNF-α, particularly during developmental periods, has been reported to modulate central immune responses in unpredictable ways and may lead to paradoxical neuroinflammatory or neurobehavioral outcomes [19,20].

In light of these considerations, the effects of IFX on the central nervous system in the context of ASD remain insufficiently characterized. In particular, studies simultaneously examining the impact of IFX treatment on behavioral outcomes, proinflammatory cytokine profiles, and regional glial activation in the hippocampus and cerebellum within a PPA-induced experimental ASD model are limited. In the present study, this PPA-induced experimental autism-like condition is referred to as the “ASD model” for clarity. Therefore, the present study aimed to comprehensively evaluate the effects of IFX treatment on behavioral performance, neuroinflammatory markers, and glial activation within this experimental ASD model.

## 2. Materials and Methods

### 2.1. Ethical Approval and Animal Welfare

All experimental procedures were approved by the Van Yüzüncü Yıl University Animal Experiments Local Ethics Committee (Approval No: 2025/11-24; 30 October 2025). The study was conducted in accordance with national regulations governing the care and use of laboratory animals and complied with the ARRIVE (Animal Research: Reporting of In Vivo Experiments) guidelines. All efforts were made to minimize animal suffering and to reduce the number of animals used throughout the experimental procedures.

### 2.2. Experimental Animals

A total of 32 male Wistar albino rats (3–5 weeks old; 125–175 g) were used in this study. Animals were obtained from the Experimental Medicine Application and Research Center of Van Yüzüncü Yıl University (YÜDETAM). Rats were housed under standard laboratory conditions (12 h light/dark cycle; 24 ± 3 °C) with free access to standard pellet chow and water. All animals were allowed an acclimatization period before the initiation of experimental procedures.

### 2.3. Drugs and Chemicals

Propionic acid (≥99.5% purity; Art. No. 6026.1; CAS No. 79-09-4) was purchased from Carl Roth GmbH + Co. KG (Karlsruhe, Germany). Infliximab (IFX) was obtained as a European Pharmacopoeia reference standard (Infliximab CRS; EDQM, Strasbourg, France; catalog code: Y0002047). The reference standard was reconstituted and diluted in sterile physiological saline immediately before intraperitoneal administration. Ketamine (Keta-Control^®^, Arion, Istanbul, Turkey) and xylazine hydrochloride (Rompun^®^ 2%, Bayer, Leverkusen, Germany) were used for anesthesia. All solutions were freshly prepared immediately before use.

### 2.4. Experimental Design and Groups

Animals were randomly assigned to four groups (*n* = 8 per group) as follows:

Randomization was performed using a simple random allocation method to minimize selection bias.

1. Control group: Rats received 0.5 mL of phosphate buffer (0.2 M, pH 7.2) via oral gavage for three consecutive days. From day 4 onward, 0.1 mL of saline was administered intraperitoneally (i.p.) once weekly for five weeks.
2. Autism (PPA) group: To induce the experimental ASD model, rats were administered propionic acid (PPA) at a dose of 250 mg/kg, dissolved in 0.2 M phosphate buffer (pH 7.2), via oral gavage for three consecutive days [21]. From day 4 onward, animals received 0.1 mL of saline i.p. once weekly for five weeks.
3. Autism + IFX group: Rats in which the experimental ASD model was induced with PPA, as described for Group 2, were treated with infliximab (IFX) at a dose of 5 mg/kg administered i.p. once weekly for five weeks, starting from day 4 following PPA administration [22,23].
4. IFX group: Rats received 0.5 mL of phosphate buffer (0.2 M, pH 7.2) via oral gavage for three consecutive days. From day 4 onward, infliximab was administered at a dose of 5 mg/kg i.p. once weekly for five weeks [22,23].

The selected dose of infliximab (5 mg/kg) was based on previous experimental studies demonstrating its anti-inflammatory efficacy in rodent models [22,23].

Animals were housed in groups under standard laboratory conditions. To prevent potential confounding effects arising from inter-group social interactions, each cage contained animals belonging to the same experimental group.

### 2.5. Behavioral Assessments

Cognitive function related to learning and memory was assessed using the Morris Water Maze test [5,24]. Social recognition, social affiliation, and social memory were evaluated by the direct social interaction test [3]. Social novelty preference was examined using the three-chamber sociability test [25]. Behavioral tests were conducted during the last 10 days of the experimental period. The direct social interaction test and the three-chamber sociability test were performed every other day at 08:00 a.m., while the Morris water maze (MWM) test was conducted daily at 16:00. All tests were applied to the same cohort of animals under standardized conditions in order to ensure reliable and comparable results across experimental groups. All behavioral assessments were performed by an investigator blinded to group allocation to minimize observer bias. All behavioral tests were conducted under controlled environmental conditions, including consistent lighting, temperature, and minimal noise levels. Before each behavioral test, animals were allowed a brief habituation period in the testing apparatus to minimize novelty-induced stress and ensure reliable behavioral responses. The sequence of behavioral testing was arranged such that social behavior assessments were performed in the morning, followed by learning and memory evaluation in the afternoon. This scheduling allowed the distribution of behavioral procedures across different time periods of the day, providing sufficient recovery time between tests and minimizing potential stress-related influences.

Repeated behavioral assessments were incorporated as part of the experimental design, with the aim of balancing test exposure across groups. While social behavior tests were conducted on five occasions (every other day), the Morris water maze was performed daily for 10 consecutive days. This approach ensured consistent behavioral engagement and enhanced comparability across experimental groups.

Behavioral tests were conducted following the initiation of the treatment protocol, and all assessments were performed after the administration of the respective agents on each testing day. This approach was intended to ensure that animals were evaluated under treatment conditions.

#### 2.5.1. Morris Water Maze Test

The Morris Water Maze (MWM) test was used to assess spatial learning and memory functions associated with the hippocampus and prefrontal cortex. The test was conducted in a circular pool (155 cm in diameter) divided into four equal quadrants (north, south, east, and west), with external visual cues placed around the tank to facilitate spatial orientation. A hidden platform was positioned in the northwest quadrant, 2 cm below the water surface, and its location remained constant throughout the training period. Rats were released from different quadrants in a pseudo-random manner and allowed a maximum of 60 s to locate the platform. During the first training day, animals that failed to reach the platform within this period were gently guided to the platform and allowed to remain there for 15 s to facilitate learning. In subsequent days, animals that failed to locate the platform within 60 s were assigned a maximum escape latency of 60 s [26].

Each rat underwent four trials per day for 10 consecutive days. Escape latency was recorded as the primary outcome measure. After each trial, animals were dried and returned to their home cages. Water temperature was maintained at 22 ± 2 °C throughout the experiment.

#### 2.5.2. Direct Social Interaction Test

The direct social interaction test was performed in an open-top Plexiglas arena measuring 40 × 40 cm. Two unfamiliar male rats were placed simultaneously in opposite corners of the arena and allowed to interact freely for 7 min. During the test period, the frequency of sniffing and grooming behaviors directed toward the conspecific was recorded. Prior to testing, animals were allowed a brief habituation period in the apparatus to minimize novelty-induced stress [3].

The test was conducted every other day during the final 10 days of the experimental period, resulting in a total of five sessions. Behavioral outcomes were calculated as the mean values obtained across these sessions. All behavioral assessments were performed by an investigator blinded to group allocation to minimize observer bias. The arena was thoroughly cleaned with 70% ethanol and dried between trials to eliminate olfactory cues. All tests were conducted under controlled environmental conditions with consistent lighting and minimal noise.

#### 2.5.3. Three-Chamber Sociability and Social Novelty Test

To assess sociability and social novelty preference, a three-chamber apparatus consisting of three adjacent Plexiglas compartments (each measuring 40 × 40 × 40 cm) with open tops and interconnected doorways was used [27,28]. Prior to testing, animals underwent a habituation period in the apparatus as described for the direct social interaction test.

During the sociability phase, an empty wire cage was placed in one side chamber, while an unfamiliar rat from a different experimental group, matched for age and sex, was placed inside a wire cage in the opposite chamber. The test rat was positioned in the central chamber and, after opening the doors, was allowed to freely explore all compartments for 10 min. The frequency of sniffing directed toward the empty cage and the unfamiliar rat was recorded. A higher frequency of sniffing toward the unfamiliar rat compared to the empty cage was considered indicative of sociability [27].

During the social novelty phase, a familiar rat from the same experimental group as the test animal and an unfamiliar rat from a different experimental group, matched for age and sex, were placed inside wire cages located in the side chambers. Care was taken to prevent the test rat from observing the placement of the animals. After placement in the central chamber, the test rat was allowed to freely explore the apparatus for 10 min. The frequency of sniffing directed toward the familiar and unfamiliar rats was recorded, and a higher frequency of sniffing toward the unfamiliar rat was interpreted as social novelty preference [28,29].

Both phases were conducted every other day during the final 10 days of the experiment, for a total of five sessions. Behavioral outcomes were calculated as the mean values obtained across these sessions.

All behavioral assessments were performed by an investigator blinded to group allocation to minimize observer bias. The apparatus was thoroughly cleaned with 70% ethanol and dried between trials to eliminate olfactory cues. All tests were conducted under controlled environmental conditions with consistent lighting and minimal noise.

### 2.6. Assessment of Biochemical Markers

At the end of the experimental period, rats were deeply anesthetized with ketamine (70 mg/kg) and xylazine (10 mg/kg) and euthanized by exsanguination. Blood samples were collected via cardiac puncture and centrifuged to obtain serum. Brains were rapidly excised and separated into right and left hemispheres. Hippocampal and cerebellar tissues from the left hemisphere were isolated and homogenized in ice-cold 0.1 M phosphate buffer (pH 7.4) to prepare 10% (*w*/*v*) tissue homogenates. The homogenates were centrifuged at 4000 rpm for 10 min at 4 °C, and the supernatants were collected for biochemical analyses [3].

Levels of BDNF, TNF-α, IL-1β, and IL-6 in serum, hippocampal, and cerebellar samples were quantified using commercially available enzyme-linked immunosorbent assay (ELISA) kits according to the manufacturers’ instructions (BDNF: RE2848R, Bioassay Technology Laboratory, Shanghai, China; TNF-α: FY-ER1407, Fine Biotech, Wuhan, China; IL-1β: FY-ER1358, Fine Biotech, Wuhan, China; IL-6: SEA079Ra, Cloud-Clone Corp., Houston, TX, USA). All samples were analyzed in duplicate. Absorbance was measured at 450 nm using a microplate reader (Multiskan SKY Microplate Spectrophotometer, Thermo Fisher Scientific, Vantaa, Finland) [30].

### 2.7. Histopathological Analysis

At the end of the experimental period, rats were euthanized, and brain tissues were carefully excised. The cerebellum was dissected from the brainstem and olfactory connections. Hippocampal and cerebellar tissues obtained from the right hemisphere were fixed in 10% neutral buffered formalin for histopathological and immunohistochemical analyses. After fixation, tissues were processed using standard histological procedures and embedded in paraffin. Serial sections of 5 μm thickness were cut from the paraffin blocks and mounted on glass slides for subsequent analyses. Sections obtained from the hippocampus and cerebellum were stained with hematoxylin–eosin (H&E) to evaluate general tissue architecture [31]. Histopathological examinations were conducted under a light microscope, and structural differences between groups were evaluated qualitatively. Evaluation of Purkinje cells in the cerebellum was based on the continuity of the Purkinje cell layer, cellular alignment, and morphological characteristics. Features such as cellular rarefaction, disruption of layer integrity, cellular shrinkage, and alterations in nuclear morphology were identified and compared across experimental groups. Histopathological examination was independently performed by two blinded observers, and consensus scores were recorded to minimize observer bias. Histopathological evaluation were scored as low (+), moderate (++), or strong (+++), and comparisons were made among experimental groups.

### 2.8. Immunohistochemical Evaluation

Immunohistochemical staining was performed to assess microglial and astrocytic activation [32,33,34]. Paraffin-embedded tissues were sectioned at 5 μm thickness and mounted onto slides coated with poly-L-Lysin. After the slides were deparaffinized and dehydrated, they were passed through xylene and an alcohol series. The slides were incubated in 3% Hydrogen peroxide (H_2_O_2_) to prevent endogenous peroxidase activity. To avoid antigen masking in the nucleus, the slides were heated in the microwave oven in antigen retrieval (citrate buffer, pH 6.1) twice for 5 min. To block nonspecific background staining, the slides were incubated in Ultra V Block for 10 min at room temperature. Sections were incubated with CD11 and GFAP primary antibodies at +4 °C in a humidity chamber overnight. Following washing the sections with PBS, Biotinylated Goat Anti-Polyvalent and Streptavidin–peroxidase conjugate was dropped and they were incubated for 10 min, respectively. Diaminobenzidine (DAB) was used as a chromogen and afterwards counterstained with Mayer’s hematoxylin. It was then viewed under a light microscope (Olympus BX53, Olympus Corporation, Tokyo, Japan), and images were analyzed and captured using cellSens software (Olympus Corporation, Tokyo, Japan). For immunohistochemical evaluation, a mean of 15–20 areas per animal was examined by random sampling in each group. It was assessed according to the density indices of the brown colour of randomly selected areas. Semi-quantitative immunohistochemical scoring was independently performed by two blinded observers, and consensus scores were recorded to minimize observer bias. Immunoreactivity levels were scored as low (+), moderate (++), or strong (+++), and comparisons were made among experimental groups.

### 2.9. Statistical Analysis

Statistical analyses were performed using SPSS software (version 21.0; IBM Corp., Armonk, NY, USA). Data are presented as mean ± standard deviation (SD). The normality of data distribution was assessed using the Shapiro–Wilk test, and homogeneity of variances was evaluated using Levene’s test.

For behavioral outcomes, including the direct social interaction test, and three-chamber sociability as well as biochemical parameters assessed at a single time point, differences among groups were analyzed using one-way analysis of variance (ANOVA), followed by Tukey’s post hoc test. In the Morris water maze test, escape latency values were analyzed separately for each training day. Between-group comparisons on the same training day were performed using one-way ANOVA followed by Tukey’s post hoc test. A *p* value < 0.05 was considered statistically significant for all analyses.

## 3. Results

### 3.1. Morris Water Maze Task

**Figure 1:**
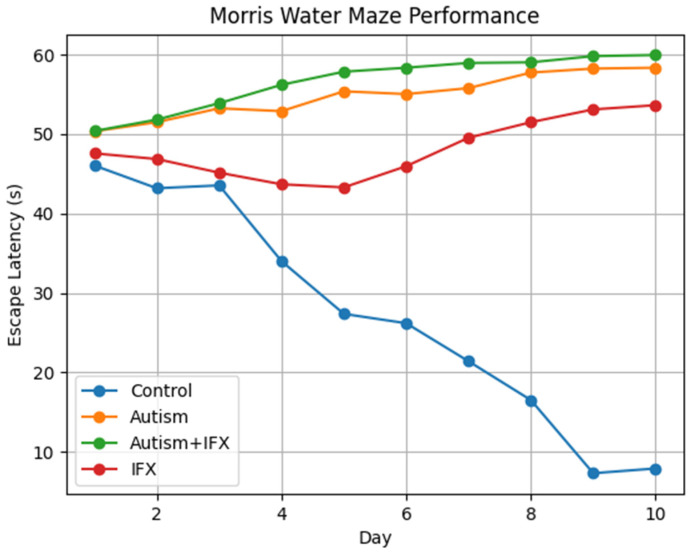
Effects of infliximab on spatial learning and memory in the Morris Water Maze test.

Spatial learning and memory were assessed by monitoring escape latency to the hidden platform throughout the final 10 days of the Morris Water Maze task (Table 1, Figure 1). Control animals showed a clear learning curve, characterized by a progressive reduction in escape latency across testing days, with significantly shorter latencies observed from day 4 onward compared with day 1 (*p* < 0.05). By the final day, the control group achieved the fastest platform acquisition among all groups. In contrast, PPA-treated rats displayed marked impairments in spatial learning. Although escape latency values were comparable to controls during the initial two days (*p* > 0.05), a significant divergence emerged from day 3 onward, with autism-model animals exhibiting persistently prolonged latencies that progressively increased over the testing period (*p* < 0.05). This deficit remained evident on day 10, indicating a pronounced disruption of spatial learning and memory retention. Administration of IFX to PPA-treated rats did not improve task performance. Escape latency values in the Autism + IFX group remained consistently elevated and closely paralleled those observed in the autism group throughout the experiment, with no significant differences detected between these groups at any time point (*p* > 0.05). In addition, latency values in this group increased significantly after day 4 compared with baseline measurements (*p* < 0.05). Rats receiving infliximab alone also exhibited impaired performance relative to controls. Although escape latencies in the IFX group were significantly longer than those of the control group from day 4 onward (*p* < 0.05), they remained significantly shorter than those recorded in the autism and Autism + IFX groups (*p* < 0.05). The IFX-treated group demonstrated an intermediate profile, with latency values higher than the control group but significantly lower than the Autism and Autism + IFX groups on most testing days, indicating a moderate impairment that does not reach the severity observed in the ASD model.

Escape latency to reach the hidden platform was recorded over 10 consecutive days. Control animals exhibited a progressive decrease in latency, indicating normal learning ability. In contrast, Autism and Autism + IFX groups showed persistently elevated latency values throughout the testing period, reflecting impaired spatial learning and memory. The IFX-treated group demonstrated an intermediate profile, with latency values higher than the control group but significantly lower than the Autism and Autism + IFX groups on most testing days, indicating a moderate impairment that does not reach the severity observed in the ASD model (Figure 1).

### 3.2. Effects on Direct Social Interaction Test

Direct social interaction was assessed by quantifying sniffing and grooming behaviors during the final five days of the experiment (Table 2). Sniffing behavior differed significantly among groups (*p* < 0.05), with PPA-treated animals exhibiting a marked reduction relative to controls. Infliximab administration did not normalize this deficit, as sniffing frequency in the Autism + IFX group remained comparable to that observed in the autism group and significantly lower than control values (*p* < 0.05). Rats receiving infliximab alone displayed intermediate sniffing activity, which was reduced compared with controls but remained higher than that observed in PPA-treated groups (*p* < 0.05).

Grooming behavior also showed significant group-dependent differences (*p* < 0.05). Control animals exhibited higher grooming frequencies, whereas grooming activity was uniformly reduced in the autism, Autism + IFX, and IFX groups, with no significant differences detected among these three groups (Table 2).

### 3.3. Three-Chamber Sociability Test

#### 3.3.1. Social Novelty Test

Social novelty was evaluated by quantifying sniffing behavior directed toward unfamiliar and familiar conspecifics (Table 3). Sniffing toward the unfamiliar rat differed significantly among groups (*p* < 0.05). Control animals exhibited a strong preference for the unfamiliar conspecific, whereas this response was markedly reduced in PPA-treated rats. Infliximab administration did not restore social novelty preference, as the Autism + IFX group showed the lowest levels of investigation toward the unfamiliar rat. Animals treated with infliximab alone displayed partially preserved novelty-related sniffing, with values intermediate between control and autism groups.

In contrast, sniffing behavior directed toward the familiar rat showed an inverse pattern (*p* < 0.05). The Autism + IFX group demonstrated the highest level of investigation toward the familiar conspecific, while control animals showed minimal interest. Sniffing frequencies toward the familiar rat in the autism and IFX groups were elevated compared with controls but did not differ significantly from each other (Table 3).

#### 3.3.2. Sociability Test

Sociability was assessed by comparing sniffing behavior directed toward an empty object and an unfamiliar conspecific (Table 4). Investigation of the empty object differed significantly among groups (*p* < 0.05). PPA-treated animals showed increased object-oriented sniffing relative to controls, with the highest levels observed in the Autism + IFX group, whereas control animals displayed minimal interest in the empty object.

In contrast, sniffing behavior directed toward the unfamiliar animal was significantly reduced in PPA-treated rats compared with controls (*p* < 0.05). Infliximab administration did not restore sociability-related investigation, as sniffing levels toward the unfamiliar conspecific in the Autism + IFX group remained comparable to those observed in the autism group. Rats receiving infliximab alone exhibited higher levels of investigation toward the unfamiliar animal than PPA-treated groups but remained below control values (Table 4).

### 3.4. Biochemical Findings

#### 3.4.1. Hippocampal Biochemical Parameters

Hippocampal levels of BDNF and proinflammatory cytokines differed significantly among groups (*p* < 0.05; Table 5). PPA exposure was associated with increased BDNF levels compared with control animals (*p* < 0.05), with the most pronounced elevation observed in the Autism + IFX group. BDNF concentrations in the autism and IFX-only groups were comparable (*p* > 0.05) and remained significantly higher than those of the control group (*p* < 0.05). Hippocampal IL-1β levels were significantly elevated in PPA-treated rats relative to controls (*p* < 0.05) and reached the highest values in the Autism + IFX group. Although infliximab alone partially reduced IL-1β levels compared with the autism group (*p* < 0.05), concentrations remained significantly higher than control values (*p* < 0.05).

In contrast, TNF-α levels showed a marked response to infliximab. PPA administration resulted in elevated hippocampal TNF-α levels compared with controls (*p* < 0.05), whereas infliximab treatment significantly reduced TNF-α concentrations in the Autism + IFX group relative to the autism group (*p* < 0.05), yielding values comparable to those observed in control animals (*p* > 0.05). TNF-α levels in the IFX-only group did not differ significantly from those of the control or Autism + IFX groups (*p* > 0.05). Hippocampal IL-6 levels were significantly increased following PPA exposure (*p* < 0.05). IL-6 concentrations were further elevated in the Autism + IFX group compared with the autism group (*p* < 0.05), while the highest IL-6 levels were detected in rats treated with infliximab alone (*p* < 0.05; Table 5).

#### 3.4.2. Cerebellar Biochemical Parameters

Cerebellar levels of BDNF and inflammatory cytokines differed significantly among groups (*p* < 0.05; Table 6). PPA exposure was associated with elevated BDNF levels relative to controls (*p* < 0.05), with the most pronounced increase observed in the Autism + IFX group. BDNF concentrations in the autism and IFX-only groups were comparable to each other (*p* > 0.05) and remained significantly higher than control values (*p* < 0.05).

Cerebellar IL-1β levels were increased in PPA-treated animals (*p* < 0.05) and were further elevated in both the Autism + IFX and IFX groups compared with the autism group (*p* < 0.05). No significant difference was detected between these two infliximab-treated groups (*p* > 0.05).

In contrast, TNF-α levels were significantly reduced following infliximab administration. While PPA treatment resulted in elevated cerebellar TNF-α levels compared with controls (*p* < 0.05), both the Autism + IFX and IFX-only groups exhibited significantly lower TNF-α concentrations than the autism group (*p* < 0.05), with no significant difference between these two groups (*p* > 0.05).

IL-6 levels displayed a divergent response pattern. The highest cerebellar IL-6 concentrations were observed in the Autism + IFX group (*p* < 0.05). In contrast, infliximab alone partially reduced IL-6 levels compared with the autism group (*p* < 0.05), although values remained significantly higher than those of controls (*p* < 0.05; Table 6).

#### 3.4.3. Serum Biochemical Parameters

Serum levels of BDNF and inflammatory cytokines differed significantly among groups (*p* < 0.05; Table 7). PPA exposure was associated with a significant increase in serum BDNF levels compared with controls (*p* < 0.05), with the most pronounced elevation observed in the Autism + IFX group. In contrast, BDNF concentrations in the IFX-only group were comparable to those of control animals (*p* > 0.05) and remained significantly lower than those detected in the autism and Autism + IFX groups (*p* < 0.05).

Serum IL-1β levels were significantly elevated following PPA administration compared with controls (*p* < 0.05) and reached maximal values in the Autism + IFX group. Although infliximab alone significantly reduced IL-1β levels relative to the autism and Autism + IFX groups (*p* < 0.05), concentrations remained higher than control values (*p* < 0.05).

A distinct pattern was observed for TNF-α. Serum TNF-α levels were increased in the autism group compared with controls (*p* < 0.05), whereas infliximab treatment significantly reduced TNF-α concentrations in the Autism + IFX group to levels comparable with those of the control group (*p* > 0.05). In the IFX-only group, TNF-α levels tended to be lower than those in the autism group; however, this difference did not reach statistical significance (*p* > 0.05). A significant difference was detected between the IFX-only and Autism + IFX groups (*p* < 0.05).

Serum IL-6 levels were significantly elevated in the autism group relative to controls (*p* < 0.05) and were further increased in the Autism + IFX group, which exhibited the highest IL-6 concentrations among all groups (*p* < 0.05). Infliximab alone resulted in intermediate IL-6 levels that remained significantly higher than those of the control group (*p* < 0.05) but lower than those observed in the autism group (*p* < 0.05; Table 7).

### 3.5. Histopathological Findings

Histopathological examinations of the hippocampal and cerebellar tissues revealed marked morphological differences among the groups.

#### 3.5.1. Hippocampus Histopathological Findings

**Figure 2:**
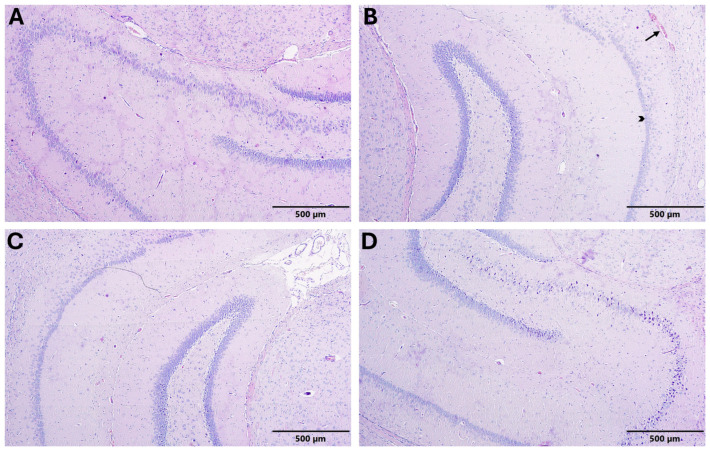
Histopathological evaluation of hippocampal tissue in experimental groups (H&E, 100×; scale bar = 500 µm). (**A**) Control group; (**B**) ASD model group (arrow indicates vascular congestion; arrowhead indicates necrotic cells); (**C**) ASD + IFX group; (**D**) IFX group.

In the control group, hippocampal layer organization was preserved. Neurons exhibited normal morphology, and neuronal integrity and cellular density appeared within normal limits. No pathological findings were observed (Figure 2A) (Table 8).

In the autism group marked vascular congestion (arrow) was observed within the blood vessels. Necrotic cells (arrowhead) were detected in the hippocampal layers. Disruption of neuronal integrity, cellular degeneration, and disorganization of lamination were evident (Figure 2B) (Table 8).

In the Autism + IFX group, vascular congestion and necrotic cells were observed. Disorganization of lamination and degenerative neuronal changes were evident, indicating a moderate level of histopathological damage (Figure 2C) (Table 8).

In the IFX group, hippocampal lamination was generally preserved, and no prominent structural disruption was observed. However, focal mild vascular congestion was noted in limited areas (Figure 2D) (Table 8).

#### 3.5.2. Cerebellum Histopathological Findings

**Figure 3:**
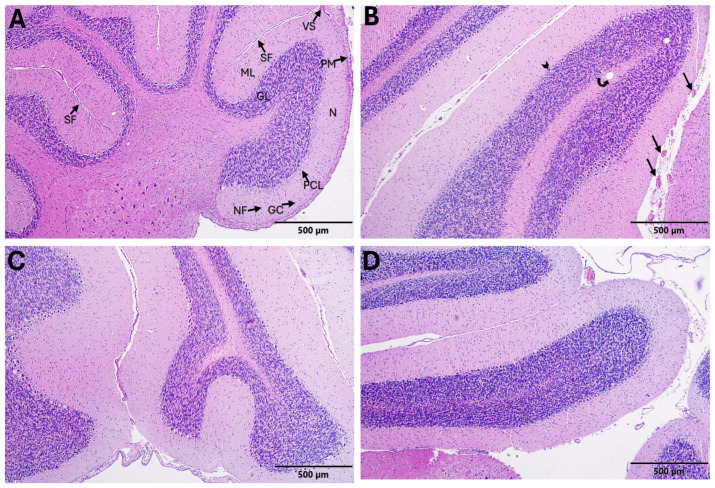
Representative histopathological images of cerebellar cortex in experimental groups (H&E, 100×; scale bar = 500 µm). (**A**) Control; (**B**) ASD model; (**C**) ASD + IFX; (**D**) IFX. Arrow indicates nuclear pyknosis; arrowhead indicates Purkinje cell depletion. ML: Molecular layer; PCL: Purkinje cell layer; GL: Granular layer; SF: Sulcus fissure; VS: Vascular structure; PM: Pia mater; N: Neuropil; NF: Nerve fibers; GC: Granule cells. Scale bar = 500 µm.

In the control group, cerebellar folia exhibited normal morphology. The molecular layer (ML), Purkinje cell layer (PCL), and granular layer (GL) were well organized and displayed normal histological architecture. Neuropil structure, vascular components, and the pia mater appeared unremarkable (Figure 3A).

In the autism group, marked neuropil disorganization and reduced cellular density were observed within the molecular layer. Increased glial cell density was evident in focal areas. The Purkinje cell layer demonstrated irregular cellular alignment, along with focal depletion and necrosis of Purkinje cells (arrowhead). Mild vacuolization and reduced cellular density were also noted in the granular layer. Vascular congestion and perivascular widening were present (Figure 3B).

In the Autism + IFX group, cerebellar alterations were present but less pronounced than those observed in the autism group. Partial improvement in Purkinje cell alignment and cellular organization was noted, although mild neuropil disorganization, vacuolization, and vascular congestion persisted (Figure 3C).

In the IFX group, cerebellar lamination was generally preserved. The ML, PCL, and GL exhibited near-normal architecture, although focal mild vascular congestion was observed (Figure 3D). The semi-quantitative histopathological scoring results are presented in Table 9.

### 3.6. Immunohistochemical Findings

#### 3.6.1. Hippocampus Immunohistochemical Findings

**Figure 4:**
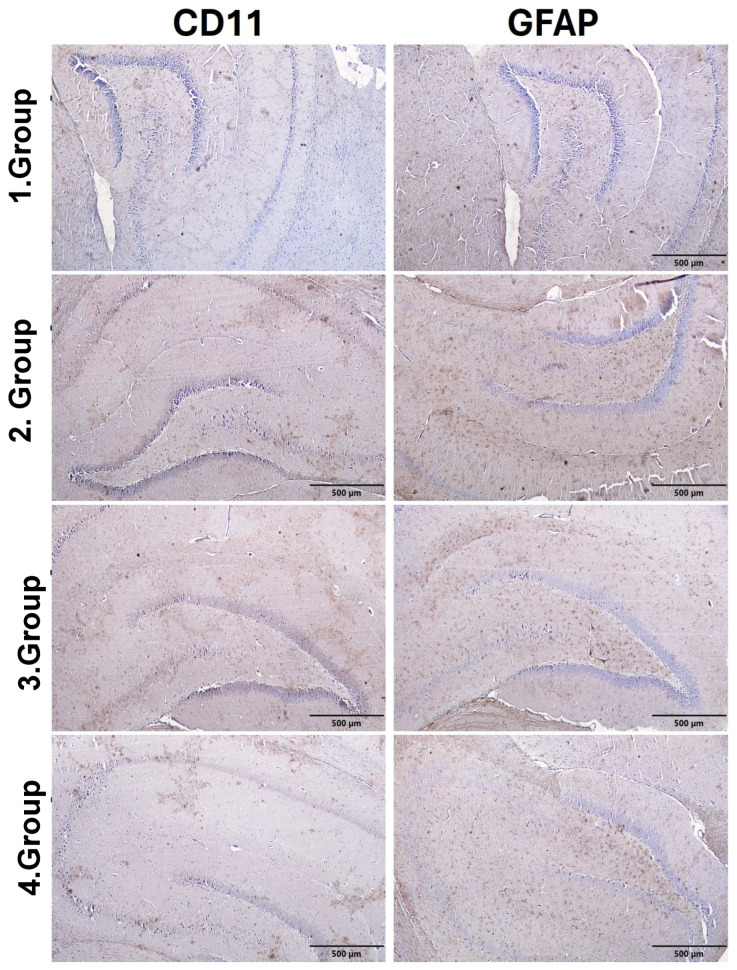
Immunohistochemical evaluation of microglial (CD11) and astrocytic (GFAP) activation in hippocampal tissue across experimental groups. Group 1: Control; Group 2: Autism; Group 3: Autism + IFX; Group 4: IFX. Scale bar = 500 µm.

The semiquantitative scores presented in Table 10 and Figure 4 revealed marked differences in CD11 and GFAP levels in hippocampal tissue among the groups. In the control group, both CD11 and GFAP exhibited low levels of immunopositivity. In the autism group, CD11 immunopositivity reached a moderate level, whereas GFAP immunopositivity reached an intense level. Similarly, in the Autism + IFX group, CD11 immunopositivity was moderate and GFAP immunopositivity was intense. In the IFX group, CD11 immunopositivity was low, while GFAP immunopositivity was detected at a moderate level. These scores indicate increased immunoreactivity in hippocampal tissue, particularly in the autism and Autism + IFX model groups.

#### 3.6.2. Cerebellum Immunohistochemical Findings

**Figure 5:**
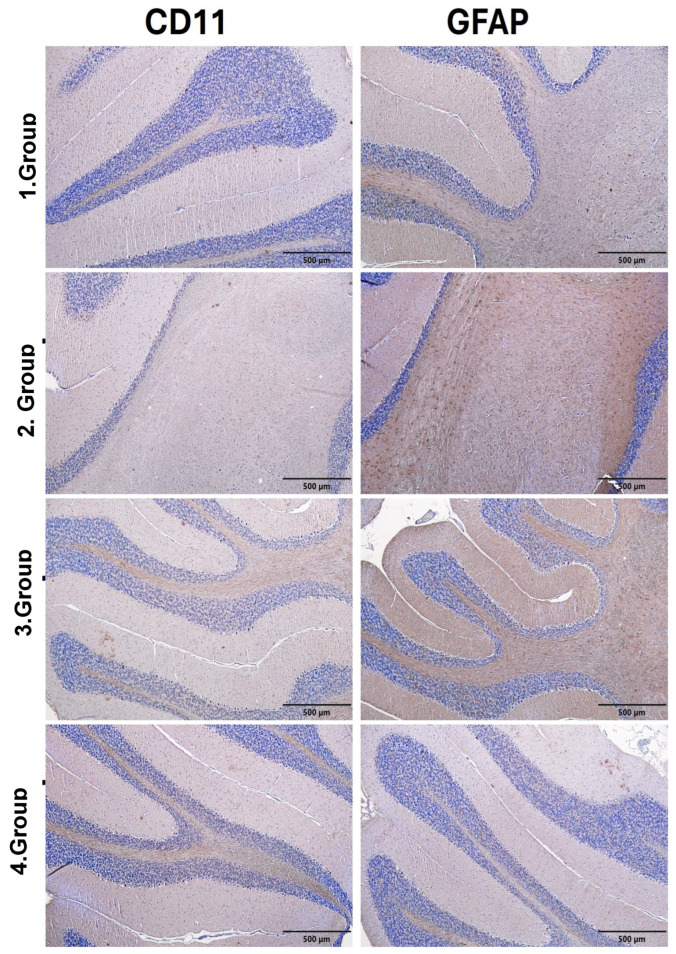
Immunohistochemical evaluation of CD11 and GFAP expression in cerebellar tissue across experimental groups. Group 1: Control; Group 2: Autism; Group 3: Autism + IFX; Group 4: IFX. Scale bar = 500 µm.

Semiquantitative scores of CD11 and GFAP immunopositivity in cerebellar tissue are presented in Table 11, and representative immunohistochemical images are shown in Figure 5. In the control group, CD11 immunoreactivity was absent, whereas GFAP immunopositivity was observed at a low level. Similarly, in the IFX group, both CD11 and GFAP immunopositivity were detected at low levels. In contrast, the autism and Autism + IFX groups exhibited moderate CD11 immunopositivity and intense GFAP immunoreactivity. Although immunoreactivity remained elevated in the Autism + IFX group, a slight reduction compared to the autism group was noted (Table 11, Figure 5).

### 3.7. Macroscopic Observations

During macroscopic examination performed at the time of brain tissue removal, limited and localized hemorrhagic areas were observed in the brains of some rats in the Autism + IFX and IFX-only treated groups. No similar macroscopic hemorrhagic foci were detected in control or autism-only groups.

Although these findings were not subjected to quantitative analysis, they may suggest that systemic TNF-α inhibition with infliximab could be associated with alterations in vascular integrity or inflammatory responses under certain conditions. However, due to the qualitative nature of these observations, no definitive conclusions can be drawn.

## 4. Discussion

This study investigated the effects of infliximab (IFX) on behavioral performance, neuroinflammatory responses, and glial activation in a propionic acid (PPA)-induced experimental ASD model. The results demonstrated that PPA exposure led to significant behavioral impairments accompanied by increased proinflammatory cytokine levels, enhanced glial activation, and structural alterations in brain tissue.

Although IFX treatment effectively reduced TNF-α levels, this reduction was not associated with improvement in behavioral outcomes. In contrast, IL-1β and IL-6 levels remained elevated, and glial activation persisted. These findings indicate that targeting TNF-α alone is insufficient to restore the complex neuroimmune balance underlying ASD pathology.

TNF-α is not only a proinflammatory cytokine but also plays essential roles in synaptic plasticity and neurodevelopment. Therefore, its non-selective inhibition during critical developmental periods may disrupt physiological neuroimmune signaling and lead to unintended functional consequences.

Notably, the concurrent persistence of increased IL-1β and IL-6 concentrations, high CD11 and GFAP immunopositivity, and deficits in social and cognitive behaviors suggests that TNF-α blockade in this ASD model gives rise to a neuroimmune response profile that diverges from the anticipated anti-inflammatory outcome. Given the complex and multifactorial nature of neuroinflammatory mechanisms involved in autism pathophysiology, these findings imply that peripheral suppression of TNF-α alone may be insufficient to restore central immune homeostasis during neurodevelopment.

TNF-α plays a central role in both neuroinflammatory processes and physiological functions such as synaptic plasticity and neurodevelopment [35]. Although TNF-α inhibitors are considered potential therapeutic approaches in ASD [36], their effects may vary depending on the developmental stage and experimental context. Clinical and experimental evidence further suggests that anti-TNF therapies may not always exert neuroprotective effects on the central nervous system. IFX administration has been associated with demyelination, oligodendrocyte damage, and multiple sclerosis–like clinical manifestations in some cases [19], as well as rare peripheral neuropathies resembling Guillain–Barré syndrome [37]. These observations indicate that although TNF-α blockade suppresses peripheral inflammation, it may influence CNS-specific immunoregulatory networks and lead to context-dependent behavioral and structural alterations.

Behavioral assessments showed that IFX treatment did not improve the impairments induced by PPA and was associated with an unfavorable behavioral profile. In the Morris Water Maze test, increased escape latency indicated deficits in spatial learning and memory, which persisted despite IFX treatment [3,4,5]. Similarly, IFX did not restore impaired social interaction and sociability behaviors, and in some cases, performance remained below that of the control group. Taken together, these findings suggest that reduction in TNF-α levels did not result in behavioral improvement and may be associated with subtle negative effects on cognitive and social functions. This supports the notion that TNF-α has complex regulatory roles in the central nervous system beyond its proinflammatory activity, and that targeting a single cytokine is insufficient to restore the complex neuroimmune balance in ASD.

Several studies in the literature have reported that infliximab can improve cognitive functions in certain pathological conditions [38,39,40]. However, these beneficial effects appear to be limited to experimental models in which neuroinflammation is predominantly of peripheral origin or in which synaptic plasticity is acutely regulated via TNF-α signaling. In contrast, in developmental neurobiological disorders such as ASD, non-selective suppression of TNF-α signaling may adversely affect learning- and memory-related processes, contrary to expectations. In this context, the present study suggests that IFX may exert context-dependent effects on neuroinflammatory structures specific to the early developmental period.

Alacabey et al. (2025) and Fyke et al. (2021) [3,41] reported that reductions in sniffing and grooming behaviors directed toward unfamiliar animals in rats subjected to experimental ASD models are associated with impaired social interaction. Similarly, in the present study, the autism group exhibited significantly reduced numbers of sniffing and grooming behaviors toward unfamiliar animals compared with the control group. The persistence of similarly low levels of these behavioral parameters in the Autism + IFX group indicates that IFX treatment did not exert a restorative effect on impaired social interaction behaviors. Moreover, the finding that sniffing and grooming frequencies toward unfamiliar animals in the IFX-only group were lower than those in the control group suggests that IFX did not produce the expected positive modulation of behavioral outcomes related to social interaction.

The three-chamber sociability test is a widely used and reliable behavioral paradigm for assessing sociability and social novelty preference in rodents [27,28]. In the literature, marked impairments in sociability and social novelty behaviors, together with reduced interest toward unfamiliar rats, have frequently been reported in PPA-induced ASD models [42,43]. Consistent with these findings, the present study demonstrated that the autism group exhibited a significant reduction in orientation toward the unfamiliar animal during both the sociability and social novelty phases. However, the observation that orientation toward the unfamiliar rat in the Autism + IFX group remained lower than that observed in the autism group indicates that IFX treatment did not exert a restorative effect on impaired sociability and social novelty behaviors. Furthermore, the finding that the number of sniffing events directed toward the unfamiliar animal in the IFX-only group was lower than that of the control group suggests that IFX did not produce the expected positive modulation of neural processes underlying social behaviors.

Elevated levels of inflammatory mediators such as TNF-α, IL-1β, IL-6, and IL-8 have been reported in the brain, cerebrospinal fluid, and serum of individuals diagnosed with ASD [28]. Similarly, experimental studies have demonstrated marked increases in the levels of proinflammatory cytokines TNF-α, IL-6, and IL-1β, which are strong indicators of immune activation, following PPA administration [6,13,44]. It has been suggested that sustained elevation of these cytokines may adversely affect neuronal maturation, differentiation, and proliferation processes, thereby contributing to neurodevelopmental disorders and behavioral abnormalities associated with ASD [45]. In the present study, consistent with the behavioral impairments observed in the ASD model, increased levels of TNF-α, IL-6, and IL-1β were detected in the hippocampus, cerebellum, and serum, suggesting a pronounced activation of neuroinflammatory processes, which represent a core pathophysiological component of ASD. This increase in proinflammatory cytokines is considered to be associated with microglial activation, compromised synaptic integrity, and disruption of neuroimmune balance. In addition, marked alterations in BDNF levels, which regulate synaptic plasticity and accompany dysregulation of the cytokine network, have also been reported in individuals with ASD [46].

BDNF is a key regulator of synaptic plasticity and neurodevelopment, and increased levels have been reported in ASD in both clinical and experimental studies [47,48,49,50,51,52,53,54,55]. In the present study, elevated BDNF levels were observed in serum, hippocampus, and cerebellum, consistent with previous findings. When considered together with increased IL-1β and IL-6 levels, these results suggest a disrupted cytokine–neurotrophin balance that may contribute to altered synaptic organization and behavioral impairments in ASD.

In the present study, although IFX significantly reduced TNF-α levels in the hippocampus and cerebellum in the ASD model, the persistence of marked increases in IL-1β and IL-6 levels suggests that anti-TNF therapy may give rise to unexpected neuroimmune consequences in developmental neurobiological structures. This finding may be related to a complex balance mechanism based on the opposing biological functions mediated by TNF-α through two distinct receptors. While TNFR1 signaling triggers apoptosis, JNK activation, increased production of reactive oxygen species, and proinflammatory cytokine release, TNFR2 generates neuroprotective signals that support neuronal survival, synaptic plasticity, and microglial homeostasis [36].

Increased IL-1β levels in the central nervous system are associated with a potent proinflammatory effect that disrupts synaptic plasticity by inhibiting long-term potentiation (LTP), enhances glutamatergic excitability, and sustains microglial activation [56,57,58]. Although IL-6 exhibits neuromodulatory functions related to learning and memory at physiological levels, elevated concentrations increase astrocytic reactivity, reduce GABAergic inhibition, and weaken synaptic integrity [59]. Indeed, experimental studies have demonstrated that learning and memory performance improves under conditions of IL-6 deficiency, whereas excessive IL-6 expression results in marked cognitive impairment [60,61]. In this context, in the present study, the persistence of elevated IL-1β and IL-6 levels despite a reduction in TNF-α following IFX treatment reveals a neuroimmune profile that is biologically consistent with the observed loss of behavioral performance.

It has been reported that IFX administration improved cognitive functions by suppressing microglial activation in an adult model of hepatic encephalopathy [40]; however, this effect is considered to be specific to conditions in which neuroinflammation develops in an ontogenetically mature brain. In contrast, in ASD—a developmental disorder—TNF-α blockade may exert different effects on synaptic pruning processes, microglial maturation, and neurotrophin balance, and may be associated with reorganization of the inflammatory microenvironment. Indeed, in the present study, the marked increase in BDNF levels following IFX administration, when evaluated together with elevated IL-1β and IL-6 levels, suggests that this response may reflect a potentially maladaptive neurotrophin profile, indicating that increased BDNF levels may not always exert neuroprotective effects within a developmental context. Although IFX has been reported to provide therapeutic benefit in certain acquired disorders [62,63], the findings of the present study appear to indicate a scenario in which IFX may disrupt the TNFR1–TNFR2 balance in neurodevelopmental structures specific to childhood, sustain glial activation, and allow the persistence of IL-1β/IL-6-mediated immuno-excitatory processes. This integrative neuroimmune framework enables a comprehensive evaluation of behavioral impairments, glial activation, and cytokine dysregulation observed in the present study.

Histopathological alterations observed in the hippocampus and cerebellum were consistent with neuroinflammatory and neurodegenerative changes in the ASD model [64]. IFX treatment did not lead to a meaningful improvement in these structural alterations, indicating limited neuroprotective efficacy.

**Figure 6:**
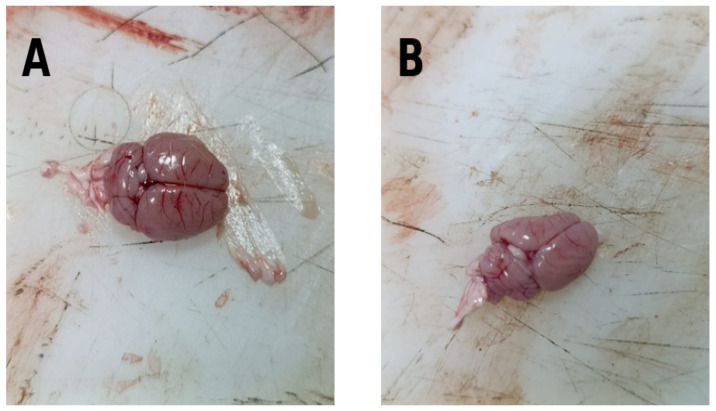
Macroscopic observations. Representative images illustrating these macroscopic findings are presented in Figure 6. (**A**) A localized and well-demarcated hemorrhagic area observed within the brain parenchyma of a rat from the Autism + IFX group. (**B**) A similarly localized macroscopic hemorrhagic focus observed in the brain of a rat from the IFX-only group. No comparable macroscopic findings were detected in the Control or Autism groups. These images represent qualitative observations and were not included in quantitative analyses.

These findings are in agreement with previous studies reporting persistent neuroinflammatory damage in PPA-induced ASD models [64]. Although IFX exerts anti-inflammatory effects, its limited impact in the present study may be related to restricted blood–brain barrier permeability or indirect peripheral-to-central immune signaling [65,66,67,68]. However, further studies are required to clarify these mechanisms. Additionally, the limited macroscopic hemorrhagic findings observed in IFX-treated groups (Figure 6), although not quantitatively analyzed, may suggest potential effects of TNF-α inhibition on neurovascular integrity during development.

The marked increase in CD11 and GFAP immunopositivity observed in the hippocampal and cerebellar tissues of the autism and Autism + IFX groups indicates that the ASD-specific pattern of glial activation was successfully reproduced in this model. Under normal conditions, TNF-α blockade has been reported to reduce microglial and astrocytic reactivity; indeed, previous studies have demonstrated that IFX can suppress neuroinflammatory responses by decreasing CD11b and GFAP expression [16,18,40]. In contrast, the persistence of high CD11 and GFAP positivity despite IFX treatment in the present ASD model suggests that TNF-α blockade may fail to elicit the expected anti-inflammatory effects within the context of developmental neuroinflammation. This finding points to the possibility that a glial reactivity profile may be maintained due to insufficient activation of TNFR2-mediated neuroprotective signaling, attenuation of microglial regulatory mechanisms, and continued IL-1β-/IL-6-driven positive feedback loops; however, direct validation of these mechanisms would require further receptor-specific and cell-level investigations.

The absence of direct evaluation of TNFR1- and TNFR2-specific signaling pathways represents a limitation of the present study. Future investigations incorporating receptor-specific molecular analyses would provide deeper insight into developmental TNF signaling and neuroimmune regulation. In addition, protein-level analyses such as Western blot to evaluate key signaling molecules were not included in the present study. Incorporating such approaches in future investigations would provide further insight into the molecular mechanisms underlying the observed effects. Furthermore, the underlying mechanisms were not directly investigated using specific inhibitors or activators of relevant signaling pathways, which limits the ability to establish causal relationships between TNF-α modulation and behavioral or molecular outcomes. In addition, histopathological and immunohistochemical evaluations were performed using semi-quantitative methods, and more advanced quantitative approaches could strengthen the interpretation of structural changes. Future studies integrating these approaches would allow a more comprehensive understanding of the complex interactions between cytokine signaling, neurotrophic factors, and behavioral outcomes.

## 5. Conclusions

In conclusion, the present study demonstrates that although IFX treatment reduced TNF-α levels in a PPA-induced ASD model, this reduction was not associated with neurobehavioral improvement; rather, it was accompanied by persistently elevated IL-1β and IL-6 levels, sustained glial activation, and impairments in cognitive and social performance. It has been proposed that non-selective suppression of TNF-α may potentially alter the balance between TNFR1- and TNFR2-mediated signaling pathways, particularly during developmental periods, thereby contributing to persistent IL-1β- and IL-6-driven neuroimmune activation. This neuroimmune profile is considered to be associated with adverse effects on mechanisms related to synaptic plasticity and neurobehavioral functions. In light of these findings, future neurodevelopmental studies should evaluate the effects of anti-TNF-α therapies not only in terms of TNF-α levels but also at the level of TNFR1- and TNFR2-specific signaling pathways, intracellular response mechanisms, and glial phenotypes. Such receptor-specific and mechanism-based approaches may help to more clearly elucidate the role of TNF-α signaling in neurodevelopmental disorders and contribute to the development of more targeted and safer immunomodulatory strategies. Furthermore, the findings indicate that while TNF-α suppression may influence cognitive function to a limited extent, it does not induce an ASD-like behavioral phenotype, highlighting the complexity of cytokine interactions during neurodevelopment.

## Figures and Tables

**Table 1:** Effects of infliximab on spatial learning and memory in PPA-treated rats.

Day	Control	Autism	Autism + IFX	IFX
1	46.04 ± 10.22 ^a^	50.36 ± 5.76 ^a^	50.39 ± 4.94 ^a^	47.57 ± 6.77 ^a^
2	43.18 ± 4.40 ^a^	51.50 ± 10.29 ^a^	51.82 ± 4.24 ^a^	46.86 ± 10.05 ^a^
3	43.54 ± 10.45 ^b^	53.25 ± 3.85 ^a^	53.89 ± 6.63 ^a^	45.14 ± 9.35 ^a,b^
4	34.00 ± 2.88 ^c,#^	52.89 ± 4.37 ^a^	56.21 ± 5.33 ^a,#^	43.68 ± 6.16 ^b^
5	27.36 ± 11.52 ^c,#^	55.39 ± 2.63 ^a^	57.86 ± 2.96 ^a,#^	43.29 ± 6.25 ^b^
6	26.18 ± 14.80 ^c,#^	55.04 ± 5.52 ^a,b^	58.36 ± 1.64 ^a,#^	45.96 ± 5.56 ^b^
7	21.39 ± 5.53 ^c,#^	55.79 ± 5.41 ^a^	58.96 ± 0.90 ^a,#^	49.54 ± 5.93 ^b^
8	16.50 ± 6.04 ^c,#^	57.75 ± 5.09 ^a,#^	59.04 ± 1.15 ^a,#^	51.50 ± 6.40 ^b^
9	7.29 ± 1.95 ^c,#^	58.25 ± 2.02 ^a,#^	59.82 ± 0.31 ^a,#^	53.11 ± 2.54 ^b^
10	7.89 ± 2.46 ^c,#^	58.36 ± 1.93 ^a,#^	59.96 ± 0.94 ^a,#^	53.64 ± 5.43 ^b^

Values sharing different superscript letters (^a^, ^b^, ^c^) within the same row indicate statistically significant differences among groups for that specific day (*p* < 0.05). Values sharing the same letter are not significantly different. The symbol (^#^) indicates a statistically significant difference compared with Day 1 within the same group (*p* < 0.05).

**Table 2:** Effects of infliximab on direct social interaction behaviors in PPA-treated rats.

Groups	Number of Sniff	Number of Grooming Events
Control	28.90 ± 2.95 ^a^	45.10 ± 3.99 ^a^
Autism	12.34 ± 1.70 ^c^	25.55 ± 5.85 ^b^
Autism + IFX	11.08 ± 2.67 ^c^	26.00 ± 6.18 ^b^
IFX	20.09 ± 7.61 ^b^	26.05 ± 3.49 ^b^

Values sharing different superscript letters (^a^, ^b^, ^c^) within the same column indicate statistically significant differences among groups (Control, Autism, Autism + IFX, IFX) (*p* < 0.05). Values sharing the same letter are not significantly different.

**Table 3:** Effects of infliximab on social novelty in PPA-treated rats.

Groups	Number of Foreign Animal Sniffs	Number of Familiar Animal Sniffs
Control	47.35 ± 3.59 ^a^	8.57 ± 0.97 ^c^
Autism	31.82 ± 1.74 ^c^	19.21 ± 4.37 ^b^
Autism + IFX	24.80 ± 3.34 ^d^	28.22 ± 5.60 ^a^
IFX	37.35 ± 0.52 ^b^	18.74 ± 3.91 ^b^

Values sharing different superscript letters (^a^, ^b^, ^c^, ^d^) within the same column indicate statistically significant differences among groups (Control, Autism, Autism + IFX, IFX) (*p* < 0.05). Values sharing the same letter are not significantly different.

**Table 4:** Effects of infliximab on sociability novelty in PPA-treated rats.

Groups	Number of Empty Object Sniffs	Number of Foreign Animal Sniffs
Control	10.25 ± 1.07 ^d^	44.90 ± 2.42 ^a^
Autism	36.97 ± 3.19 ^b^	31.32 ± 1.41 ^b,c^
Autism + IFX	43.20 ± 2.52 ^a^	28.10 ± 3.61 ^c^
IFX	29.70 ± 2.83 ^c^	33.35 ± 2.42 ^b^

Values sharing different superscript letters (^a^, ^b^, ^c^, ^d^) within the same column indicate statistically significant differences among groups (Control, Autism, Autism + IFX, IFX) (*p* < 0.05). Values sharing the same letter are not significantly different.

**Table 5:** Effects of infliximab treatment on hippocampal biochemical parameters (BDNF, TNF-α, IL-1β and IL-6) in PPA-treated rats.

	Control	Autism	Autism + IFX	IFX
BDNF (pg/mL)	521.82 ± 52.94 ^c^	810.67 ± 61.64 ^b^	904.89 ± 78.26 ^a^	777.38 ± 62.61 ^b^
IL-1β (pg/mL)	96.31 ± 1.55 ^d^	123.67 ± 10.54 ^b^	138.67 ± 5.02 ^a^	113.82 ± 7.32 ^c^
TNF-α (pg/mL)	1.81 ± 0.52 ^b^	3.14 ± 0.57 ^a^	1.24 ± 0.18 ^c^	1.56 ± 0.10 ^b,c^
IL-6 (pg/mL)	98.50 ± 6.38 ^d^	116.79 ± 9.88 ^c^	180.07 ± 17.83 ^b^	212.5 ± 4.29 ^a^

Values sharing different superscript letters (^a^, ^b^, ^c^, ^d^) within the same column indicate statistically significant differences among groups (Control, Autism, Autism + IFX, IFX) (*p* < 0.05). Values sharing the same letter are not significantly different.

**Table 6:** Effects of PPA and infliximab on cerebellar levels of BDNF and pro-inflammatory cytokines (IL-1β, TNF-α, IL-6).

	Control	Autism	Autism + IFX	IFX
BDNF (pg/mL)	560.69 ± 112.94 ^c^	874.13 ± 84.32 ^b^	1062.96 ± 224.48 ^a^	868.43 ± 35.04 ^b^
IL-1β (pg/mL)	108.65 ± 9.94 ^c^	125.68 ± 7.42 ^b^	146.59 ± 4.09 ^a^	139.02 ± 19.18 ^a^
TNF-α (pg/mL)	2.09 ± 0.53 ^b^	3.48 ± 0.29 ^a^	1.64 ± 0.64 ^b,c^	1.29 ± 0.28 ^c^
IL-6 (pg/mL)	95.32 ± 19.35 ^d^	219.12 ± 22.88 ^b^	277.19 ± 13.77 ^a^	122.111 ± 6.45 ^c^

Values sharing different superscript letters (^a^, ^b^, ^c^, ^d^) within the same column indicate statistically significant differences among groups (Control, Autism, Autism + IFX, IFX) (*p* < 0.05). Values sharing the same letter are not significantly different.

**Table 7:** Effects of PPA and infliximab on serum BDNF and inflammatory cytokines (IL-1β, TNF-α, IL-6).

	Control	Autism	Autism + IFX	IFX
BDNF (pg/mL)	391.77 ± 34.94 ^c^	568.29 ± 48.63 ^b^	636.05 ± 45.47 ^a^	421.25 ± 24.63 ^c^
IL-1β (pg/mL)	146.11 ± 9.24 ^d^	173.04 ± 2.84 ^b^	185.32 ± 2.22 ^a^	157.67 ± 6.32 ^c^
TNF-α (pg/mL)	8.25 ± 2.60 ^b^	11.07 ± 2.07 ^a^	8.27 ± 1.28 ^b^	8.74 ± 2.66 ^a,b^
IL-6 (pg/mL)	86.81 ± 3.74 ^d^	255.69 ± 33.76 ^b^	409.85 ± 77.43 ^a^	115.82 ± 8.20 ^c^

Values sharing different superscript letters (^a^, ^b^, ^c^, ^d^) within the same column indicate statistically significant differences among groups (Control, Autism, Autism + IFX, IFX) (*p* < 0.05). Values sharing the same letter are not significantly different.

**Table 8:** Semi-quantitative histopathological scoring of hippocampal tissue.

Groups	Vascular Congestion	Necrosis	Disruption of Neuronal Integrity	Cellular Degeneration
Control	−	−	−	−
Autism	+++	++	++	+++
Autism + IFX	++	+	++	++
IFX	+	−	−	−

(−): no change; (+): mild; (++): moderate; (+++): severe.

**Table 9:** Semi-quantitative histopathological scoring of cerebellar tissue.

Groups	Vascular Congestion	Necrosis	Disruption of Neuronal Integrity	Vacuolization
Control	−	−	−	−
Autism	++	++	++	++
Autism + IFX	+	+	+	+
IFX	−	−	−	−

(−): no change; (+): mild; (++): moderate.

**Table 10:** CD11 and GFAP immunopositivity scores in hippocampal tissue.

Groups	CD11	GFAP
Control	+	+
Autism	++	+++
Autism + IFX	++	+++
IFX	+	++

Scoring: + = low, ++ = moderate, +++ = intense immunoreactivity.

**Table 11:** CD11 and GFAP immunopositivity scores in cerebellar tissue.

Groups	CD11	GFAP
Control	0	+
Autism	++	+++
Autism + IFX	++	+++
IFX	+	+

Scoring: 0 = negative, + = low, ++ = moderate, +++ = intense immunoreactivity.

## Data Availability

The datasets generated and analyzed during the current study are available from the corresponding author upon reasonable request.

## Abbreviations

The following abbreviations are used in this manuscript:

ASD	Autism Spectrum Disorder
PPA	Propionic Acid
IFX	Infliximab
TNF-α	Tumor Necrosis Factor-alpha
IL-1β	Interleukin-1 beta
IL-6	Interleukin-6
BDNF	Brain-Derived Neurotrophic Factor
CNS	Central Nervous System
BBB	Blood–Brain Barrier
GFAP	Glial Fibrillary Acidic Protein
CD11	Cluster of Differentiation 11
